# Frida Kahlo (1910–1954). Self-Portrait with Monkey (1938)

**DOI:** 10.3201/eid0902.000000

**Published:** 2003-02

**Authors:** Polyxeni Potter

**Affiliations:** * Centers for Disease Control and Prevention, Atlanta, Georgia, USA

**Figure:**
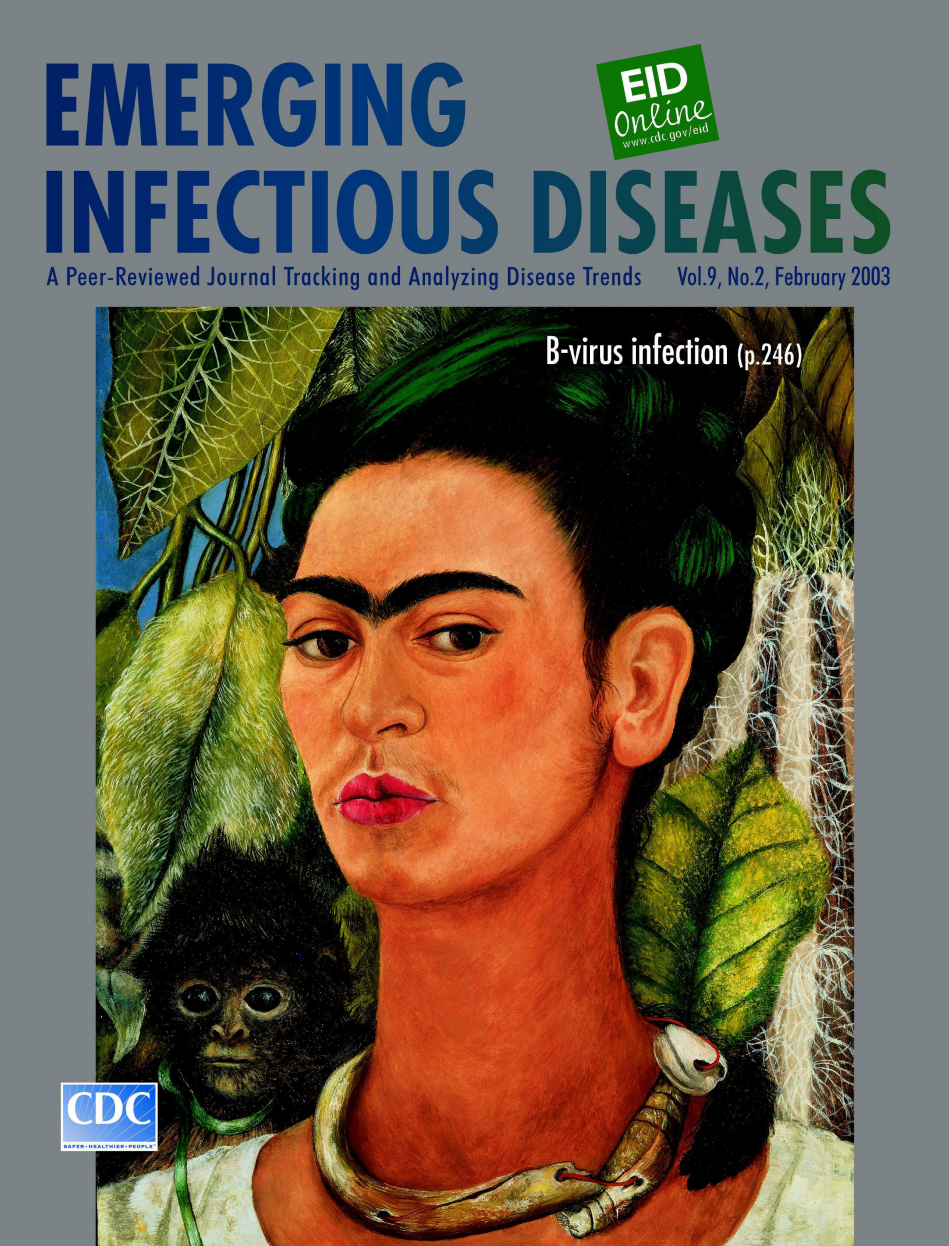
Frida Kahlo (1910–1954). Self-Portrait with Monkey (1938). Oil on masonite, 16" x 12" Albright-Knox Art Gallery, Buffalo, New York, USA Copyright 2003 Banco de México Diego Rivera & Frida Kahlo Museums Trust. Av. Cinco de Mayo No. 2, Col. Centro, Del. Cuauhtémoc 06059, México, D.F.**]**

Frida Kahlo was born in Mexico City, the third daughter of a German father and a Spanish and Native American mother. Her life was marred by physical trauma—from childhood polio that left her with a limp, to serious injury in 1926, when a bus she was riding collided with a streetcar. Lifelong pain and its psychological aftermath had a profound effect on her artistic development (*1*).

Kahlo was well educated and fiercely independent. A frail girl with a limp, she set out to be a tomboy, an intellectual, a heartbreaker, and a communist. Relentless physical pain, marital strife, and emotional rejection marked the course of her life. Her work, which incorporates Mexican folk motifs and particularly the small votive pictures known as retablos, exudes powerful feeling and is unlike that of any of her contemporary Mexican muralists (*2*). Characterized by boundless energy and strength, her paintings represent her passion for meaning and truth, feistiness and defiance of limits, intimate acquaintance with suffering, and finally poignant acknowledgment of things as they are.

Kahlo’s artistic talent was recognized by the French poet and critic André Breton in 1938, when he visited Mexico. Breton, who had studied medicine and worked on psychiatric wards during World War I, was a founder and chief promoter of the surrealist movement. The movement, partly borne of post–World War disillusionment, promoted a “revolution of the mind” against a civilization that seemed to be lowering human aspirations and proliferating human misery. Surrealism sought to synthesize humans and their world, eliminating the barriers between dream and reality, reason and madness, persons and things (*3*).

During her early association with Breton in Mexico (which he termed a “naturally surrealist country”), Kahlo worked alongside the surrealists, yet she denied any connection with them: “They thought I was a surrealist…but I wasn’t. I never painted dreams, I painted my own reality” (*2*). Even if she never espoused surrealist ideology, Kahlo seemed to embody it. She transcended her physical suffering and delved into untapped emotional depths for universal truth, which she uncovered and brought to the viewer in raw, brilliant color. In a surrealist manner, Kahlo’s work was permeated by her tempestuous life and cannot be fully understood apart from it.

From her vivid self-portrait on the cover of this month’s Emerging Infectious Diseases, Frida Kahlo casts a pensive but challenging look at a world that denied her the comforts of health. Like an exotic flower, she embellishes the luscious tropical tableau. Yet, in spite of her regal demeanor and the scene’s vibrant hues, something is troubling about the picture. Menace lurks in nature itself, which though seemingly embracing, is not unqualifiedly benign. The enigmatic presence of the monkey heightens the portrait’s uneasiness. Might it be the devil, as purported in Kahlo’s native Mexico? Non-human primates are frequent human playmates in the arts, the circus, and the streets—always amusing, romantic, and mysterious, and sometimes dark. Might the monkey on Kahlo’s back be the harbinger of ill health? Like her contemporaries, Kahlo knew little of her close phylogenetic kinship with her pet or the extreme caution prescribed by this kinship. The threat signaled by the presence of a primate, be it turbulence in Kahlo’s life or herpes B viruses in ours, remains uncharted. The monkey on our back is to decipher the zoonotic puzzle of infection that perpetuates suffering and limits the immense capacity of the human spirit.
